# A GHz Silicon-Based Width Extensional Mode MEMS Resonator with *Q* over 10,000

**DOI:** 10.3390/s23083808

**Published:** 2023-04-07

**Authors:** Wenli Liu, Yujie Lu, Zeji Chen, Qianqian Jia, Junyuan Zhao, Bo Niu, Wei Wang, Yalu Hao, Yinfang Zhu, Jinling Yang, Fuhua Yang

**Affiliations:** 1 Institute of Semiconductors, Chinese Academy of Sciences, Beijing 100083, China; 2 Center of Materials Science and Optoelectronics Engineering, University of Chinese Academy of Sciences, Beijing 100049, China; 3 State Key Laboratory of Transducer Technology, Shanghai 200050, China; 4 Kunming Institute of Physics, Kunming 650223, China

**Keywords:** MEMS resonator, width extensional mode, GHz frequency, quality factor

## Abstract

This work presents a silicon-based capacitively transduced width extensional mode (WEM) MEMS rectangular plate resonator with quality factor (*Q*) of over 10,000 at a frequency of greater than 1 GHz. The *Q* value, determined by various loss mechanisms, was analyzed and quantified via numerical calculation and simulation. The energy loss of high order WEMs is dominated by anchor loss and phonon-phonon interaction dissipation (PPID). High-order resonators possess high effective stiffness, resulting in large motional impedance. To suppress anchor loss and reduce motional impedance, a novel combined tether was designed and comprehensively optimized. The resonators were batch fabricated based on a reliable and simple silicon-on-insulator (SOI)-based fabrication process. The combined tether experimentally contributes to low anchor loss and motional impedance. Especially in the 4th WEM, the resonator with a resonance frequency of 1.1 GHz and a *Q* of 10,920 was demonstrated, corresponding to the promising *f* × *Q* product of 1.2 × 10^13^. By using combined tether, the motional impedance decreases by 33% and 20% in 3rd and 4th modes, respectively. The WEM resonator proposed in this work has potential application for high-frequency wireless communication systems.

## 1. Introduction

Wireless communication systems are developing toward large capacity, high date rate, and high-level IC integration [1,2]. Micro-electro-mechanical system (MEMS) resonators with ultra-high frequency (UHF) with a small footprint and low power consumption have emerged as a core device for wireless communication systems. In addition, MEMS resonators are produced with silicon process and, therefore, have good compatibility with ICs [3,4]. GHz MEMS resonators with high *Q* could make possible RF channel-selected filters, reducing the off-chip parts count of receivers, and thus greatly simplify the wireless communication receivers [5,6].

High resonant frequencies can be achieved with bulk modes of Si-based MEMS resonators, including Lamé mode, whispering gallery mode (WGM), width extensional mode (WEM), and so on [7,8]. The Lamé mode has a high *Q* value but transmits shear waves, and the stiffness is small; thus, is difficult to achieve ultra-high frequency. The reported maximum frequency of Lamé mode is 167 MHz [9]. WGMs have relatively low anchor loss, but their resonance frequency is hard to reach at GHz [10]. WEMs is based on longitudinal waves, high-order modes can achieve high frequencies above GHz, but the mode shape varies with the dimensions of the rectangular plate, resulting in slow optimization. This work achieved high *Q* values for high-order WEMs vibrating at GHz via optimizing the tether and resonator body.

However, it is challenging to maintain high *Q* for GHz resonators. Firstly, when the fundamental frequency reaches GHz, the feature size of resonators has to be scaled down to micro/nano-scale. The resonant frequency of the resonator is expressed in Equation (2); the high-order modes can achieve a high frequency, but as shown in Equations (1) and (4), the effective stiffness increases when the resonant frequency increases, resulting in the larger motional impedance. Secondly, in the Akhiezer regime, for a given material, the *f* × *Q* product is theoretically constant; thus, the upper limit of *Q* tends to decrease with increasing resonance frequency [11,12]. The motional impedance is large at high frequencies, and the capacitive area is hardly improved via increasing the effective stiffness. However, for the WEM, with the frequency mainly depending on width, the capacitive area can be enlarged by extending the length *L*, and the motional impedance can be expressed as Equation (4), where increasing the length *L* will reduce the motional impedance.

(1) $$k_{eff}=\omega^{2}m_{eff},$$

where *k_eff_* is the effective stiffness, *ω* is the resonance angular frequency, and *m_eff_* is the effective mass. 

In addition, MEMS UHF resonators suffer from severe anchor loss and phonon-phonon interaction dissipation (PPID). For the bulk modes, achieving ultra-high frequencies by either shrinking resonator sizes or exciting high-order modes tends to result in severer anchor loss due to the reduced nodal region, and some vibration modes could be suppressed; for example, the *Q* value of the 1st contour mode disk resonator is 23,000 at 193.2 MHz, while the 3rd mode at 829.6 MHz is undetectable [13]. In previously reported work [14], a width extensional mode (WEM) resonator was developed with straight tethers attached at the middle of the plate, corresponding to the nodal regions of the odd order mode shapes. However, for the even modes, there exist large vibration motions in the support ends, which lead to severe energy loss and undetectable *Q* values. Therefore, significant *Q* degradation at higher frequencies is a challenging issue for MEMS resonators; so far, the GHz silicon resonator with *Q* over 10,000 is seldom reported.

In this work, a GHz WEM rectangle plate resonator with a *Q* over 10,000 was developed. The dimensions of the WEM resonators are optimized to ensure the low amplitude of the coupling position between the resonator body and tether in high order. A novel combined tether is designed to attenuate severe anchor loss and large motional impedance at high frequency. A reliable SOI process was exploited to fabricate the resonators. In the 4th WEM, the frequency is 1.1 GHz, and the *Q* value reaches 10,920. The promising *f* × *Q* of 1.202 × 10^13^ is demonstrated for the capacitively transduced Si-based resonators.

## 2. Resonator Design

### 2.1. Width Extensional Mode (WEM) Resonator

**Figure 1:**
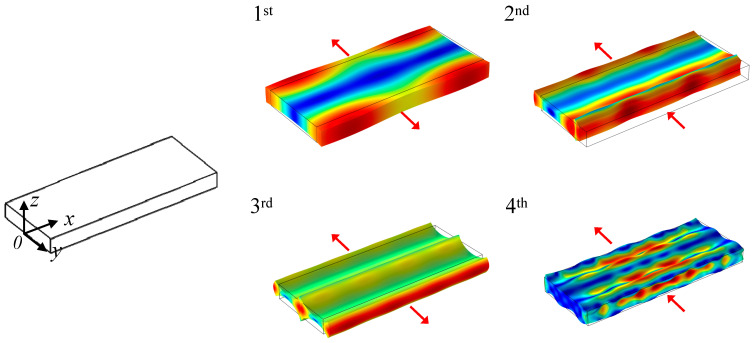
The mode shapes of the 1st to 4th WEMs. The red arrows indicate the vibration direction.

The mode shapes of rectangular plate resonators in the 1st to 4th WEMs are depicted in Figure 1. When vibrating in odd WEMs, the displacement distribution along the length (*x*-axis) of the resonator is symmetric, and the motion directions of the two half bodies of the resonator about the symmetric *x*-*y* plane are opposite, as shown in the 1st and 3rd WEMs in Figure 1. In even modes, the displacement distribution is anti-symmetric, the resonator body displaces along the same direction, as shown in the 2nd and 4th WEMs in the Figure 1. The length of the extensional modes can be regarded as the 1 D mode of longitudinal waves, and the displacement distribution follows the simple sinusoidal function [14]. For the WEMs, due to the influence of the Poisson effect, the change of length in the WEM mode will lead to the change of its displacement distribution in the *y*-axis direction, and, thus, the mode shapes of WEMs are complicated and change with the size of the rectangular plate [15]. However, this kind of change cannot be calculated by analytic expression; it needs to be obtained by the finite element analysis method.

**Figure 2:**
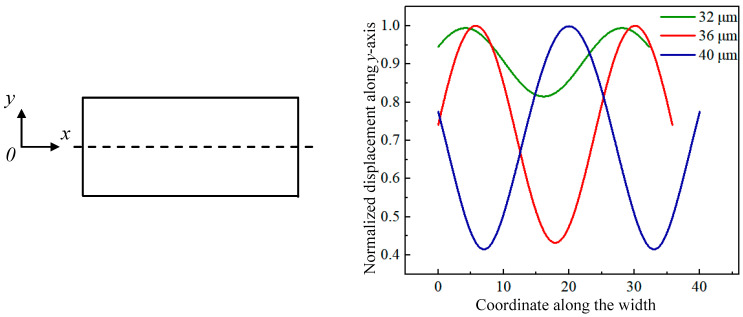
Displacement distribution along the *x*-axis for the 1st WEM of the 13.5 μm-wide resonator with lengths from 32 μm to 40 μm.

Figure 2 gives the displacement distribution along the *x*-axis for the 1st WEM resonator with a width of 13.5 μm and lengths of 32–40 μm in COMSOL Multiphysics, where the origin of the coordinate is located at the plate corner. 

In each mode of the WEM, the vibration is mainly along the width (*y*-axis), (Figure 1). The resonance frequencies of the WEMs are mainly associated with the width, and can be approximately expressed as [16]:

(2) $$f=\frac{n}{\lambda }\sqrt{\frac{E}{\rho }}=\frac{n}{2W}\sqrt{\frac{E}{\rho }},$$

where *λ* denotes the resonance wavelength, and *n* refers to the mode order. *W* is the plate width, *E* and *ρ* are the Young’s modulus and density of the material, respectively. Since the standing wave in WEMs behaves as a longitudinal wave, it is apt to achieve a higher frequency compared with the shear wave mode (e.g., Lamé mode) [15]. As described in Equation (2), high frequency can be obtained with a smaller width and higher-order vibration.

**Figure 3:**
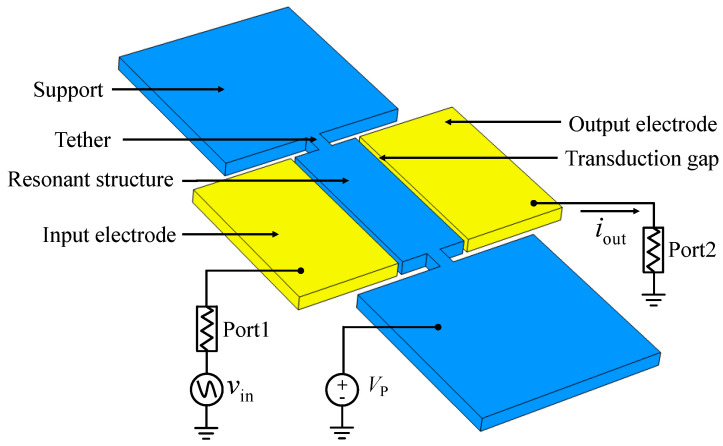
The schematic of the WEM resonator.

The schematic of the WEM resonator with a two-port measurement configuration is shown in Figure 3. Tethers are suspended in the middle of the plate width. The driving and sensing electrodes are positioned on both sides of the resonator structure, and they are separated from the resonator by electrostatic transduction gaps. The DC bias voltage is applied to the resonator, and, meanwhile, the AC voltage is provided to the driving electrode to excite the resonator. This electrode configuration avoids complicated electrode leads, and the feedthrough signal between AC signal electrodes will be minimal. The output AC current is [17]:

(3) $$i_{out}=\frac{v_{in}}{R_{x}},$$

where *v_in_* is the input AC voltage, and *R_x_* is the motional impedance of WEMs:

(4) $$R_{x}=\frac{\alpha k_{eff}g^{4}}{\omega Q\varepsilon_{{}_{0}}^{2}L^{2}h^{2}V_{{}_{p}}^{2}}=\frac{\alpha n\pi g^{4}\sqrt{\rho E}}{Q\varepsilon_{{}_{0}}^{2}LhV_{{}_{p}}^{2}},$$

where *V_P_* is the bias voltage, *g* is the size of transduction gap, *L* and *h* refer to the length and thickness of the plate, respectively, and *α* is the mode coefficient related to the mode shape. Clearly, low motional impedance is important for a large output signal. According to Equation (4), the size of the transduction gap is critical for low motional impedance, thus a spacing gap of 70 nm is designed. Besides, high *Q* values are beneficial for motional impedance reduction. In order to maintain low motional impedance and avoid charge cancellation at GHz, the thickness of the resonator is designed at 3 μm, larger than the wavelength, to avoid anti-phase vibration along the *z*-axis and an increase of the motional impedance at high frequency [15]. 

### 2.2. Quality Factor Optimization

The *Q* value is related to the energy loss of the resonator per vibration cycle, and is defined as:

(5) $$Q=2\pi \frac{E_{\mathrm{stored}}}{E_{\mathrm{loss}\;\mathrm{per}\;\mathrm{cycle}}},$$

The energy loss per cycle is subdivided into varying loss mechanisms, and the *Q* value can also be expressed as:

(6) $$\frac{1}{Q}=\sum_{i}\frac{1}{Q_{i}},$$

where *Q_i_* represents the individual loss mechanism, and the lowest *Q_i_* dominates the total *Q*. The major energy loss sources for MEMS resonators are air damping, thermal elastic damping (TED), anchor loss, and phonon-phonon interaction dissipation (PPID). The air damping is the major loss mechanism in air [18,19]. The resonator in this work is measured in vacuum, and thus the air damping can be ignored.

The TED of the resonator arises from irreversible heat flow caused by elastic deformation during vibration [20,21,22]. The thermodynamic equation of the WEM resonator is written as [23]:

(7) $$\kappa \nabla^{2}T-C_{v}\frac{\partial T}{\partial t}-\alpha (3\lambda +2\mu )T_{0}\nabla \cdot \left(\frac{\partial u}{\partial t}\right)=0,$$

where *κ, C_v_*, and *α* refer to the thermal conductivity, volumetric heat capability, and the coefficient of thermal expansion, respectively. *T*_0_ and *T* are the temperature of the ambience and solids. *λ* and *μ* denote the elastic Lamé parameters. *u* is the displacement vector. *Q_TED_* is calculated by combining Equation (7), motion Equation (8) [23], and Equation (9):

(8) $$\rho \frac{\partial^{2}u}{\partial t^{2}}=\mu \nabla^{2}(\frac{\partial u}{\partial n})+(\lambda +\mu )\nabla^{2}(\frac{\partial u}{\partial n})-\alpha (3\lambda +2\mu )\frac{\partial T}{\partial n},$$

where *n* is the *x*, *y*, *z* directions.

(9) QTED=Re(ω0)2Im(ω0),

where Re(*ω_0_*) is the intrinsic resonant frequency, and Im(*ω_0_*) is the energy loss caused by TED. For WEMs, the length and width of the plate can be optimized to make the middle of the width as a displacement node; thus, the effect of the support structure on the mode shape can be reduced and *Q*_TED_ is improved. In the first five WEMs, the *Q*_TED_ are more than 5 × 10^5^, indicating that the TED is not the dominant loss mechanism.

**Figure 4:**
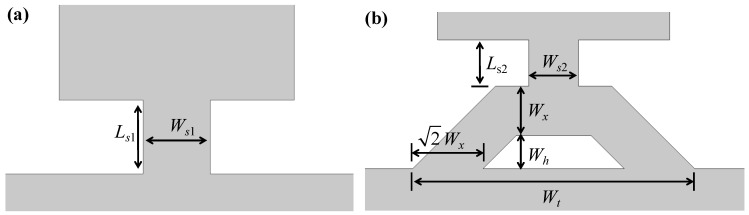
The schematic of the WEM resonators (**a**) with the straight and (**b**) with the combined tether.

The anchor loss is the acoustic energy radiated into the support base through the tether. As shown in the Equation (10) [24]:

(10) $$Q_{anchor}=2\pi (\frac{E_{res}}{E_{teth}})(\frac{E_{teth}}{E_{\mathrm{teth-loss}}})=2\pi (\frac{E_{res}}{E_{\mathrm{teth-loss}}}),$$

where *E_res_* is the energy stored in the resonator body, *E_teth_* is the energy stored in the tethers, and *E_teth-loss_* is the energy dissipated from the tether to the support base. Actually, *Q_anchor_* is determined by the ratio between *E_res_* and *E_tether-loss_*; thus, it is critical to reduce *E_tether-loss_* for enhancing *Q_anchor_*. The resonator body and support base can be regarded as a binary coupled system, and the effective stiffness of tether determines the energy transfer between the coupled resonator and tether. To optimize the energy distribution and improve *Q_anchor_*, a low-stiffness combined tether is designed, as illustrated in Figure 4b. The tether is an axisymmetric structure composed of a straight beam and an isosceles trapezoidal frame where the bottom is hollowed out. The end of the straight beam is attached to the midpoint of width, and the bottom ends of the trapezoidal frame are connected to the support base. The hollow between the combined tether and support base forms an acoustic impedance mismatch interface, which reflects part of the acoustic wave back to the resonator to further reduce the anchor loss [19].

**Figure 5:**
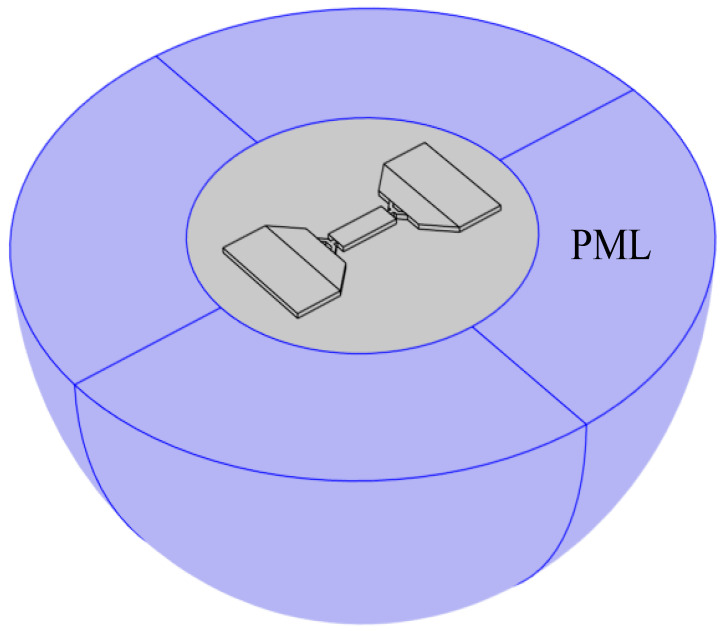
Comsol simulation of *Q_anchor_* with PML.

The *Q_anchor_* of the WEM resonators is evaluated by a COMSOL Multiphysics model shown in Figure 5. The hemispherical perfect matched layer (PML) is employed as an absorption boundary to simulate the acoustic energy dissipation in a semi-infinite support base, and the *Q_anchor_* of the resonator can be calculated as:

(11) Qanchor =Reω2Imω,

The geometry of the combined tether is comprehensively optimized by a parameter sweep to enhance *Q_acnhor_*. The WEM resonator with the combined tether is illustrated in Table 1.

Table 2 compares the simulated *Q_anchor_* of the 1st, 3rd, and 4th WEMs resonators with the combined tether and with the straight tether. For the 1st WEM, the energy discrepancy between the resonator and the combined tether is insignificant, much energy is dissipated through the tether, and the increase of the *Qanchor* is not apparent. For the 3rd and 4th modes, the stiffness of the resonator is significantly improved, and *Qanchor* values clearly rise. It should be noted that the ratios of the energy stored in tethers to the energy dissipated to the support base are various for different WEMs. The 2nd WEM is seriously distorted due to large amplitude vibration in the tether support ends.

**Figure 6:**
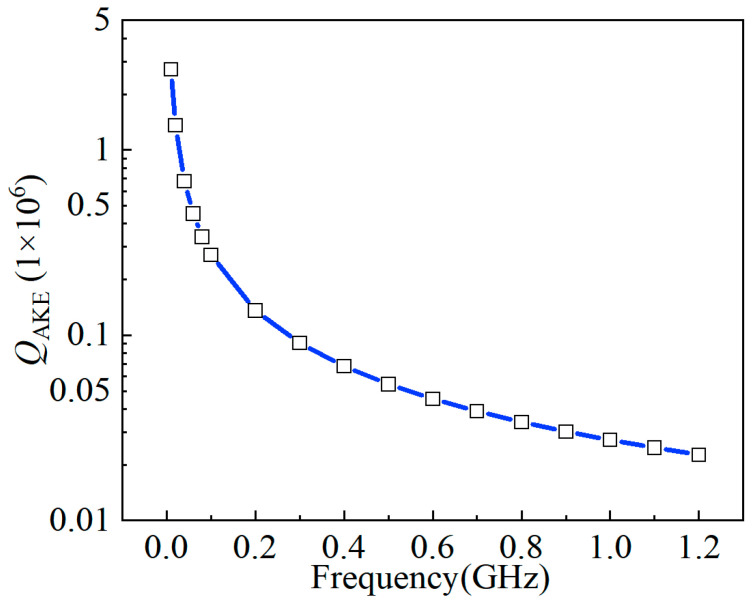
The frequency dependence of *Q_AKE_*.

The PPID comes from scattering of the acoustic phonons and determining the limit of *f × Q* product. The transition frequency from the Akhiezer (AKE) to the Landau-Rumer regime can be calculated as [25]:

(12) $$f_{\tau }=\frac{1}{2\pi \tau },$$

where *f_τ_* and *τ* are the transmition frequency and the relaxation time, respectively. For the proposed WEMs, the longitudinal acoustic wave propagates in the <110> direction in silicon; therefore, the relaxation time is 0.673 × 10^−10^ s [26]. In terms of (11), the transition frequency is estimated as 2.36 GHz. As a result, the resonator operates in the AKE regime below 1.1 GHz. The *Q_AKE_* can be evaluated by the following equation [27]:

(13) $$Q_{AKE}=\frac{\rho v_{a}^{2}}{C_{v}T\gamma_{eff}{}^{2}}\frac{1+{\left(\omega \tau \right)}^{2}}{f\tau_{}},$$

where *v_a_*, *C_v_*, *τ* and *γ_eff_* denote the acoustic velocity, volumetric heat capability, relaxation time, and effective Grüneisen parameter, respectively. For the longitudinal wave mode, *γ_eff_* is 0.51 [28]. The frequency dependence of *Q_AKE_* is plotted in Figure 6 pursuant to (13). As can be seen, the *Q_AKE_* goes down to 3 × 10^4^ at a frequency near 1 GHz, indicating that the AKE damping is the dominant loss.

## 3. Fabrication Process

**Figure 7:**
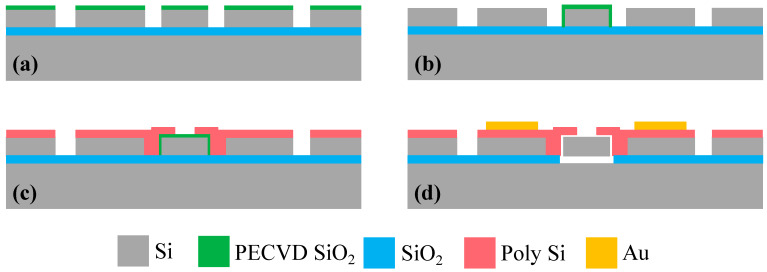
Process flow for the WEM resonators: (**a**) SOI wafer with a PECVD SiO_2_ layer deposited as a mask, (**b**) the thermal oxide layer grown on the SOI wafer by dry etching, (**c**) the deposition of LPCVD polysilicon to form AC signal electrodes and (**d**) the deposition of thick Au/Cr electrode pads by e-beam evaporation and lift-off process.

**Figure 8:**
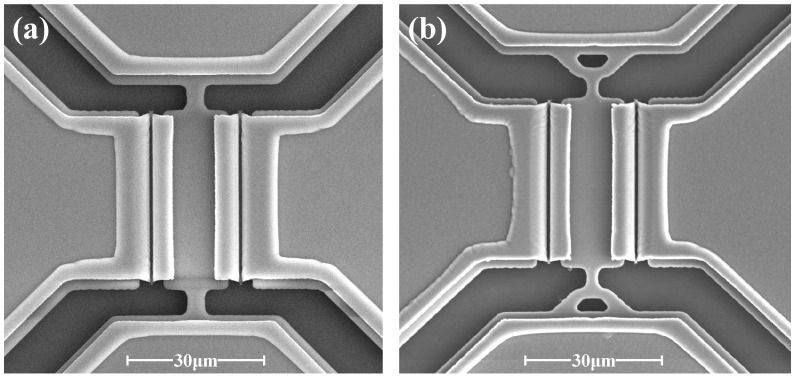
SEM photographs of the WEM resonators supported by (**a**) the straight tethers and (**b**) the combined tethers.

A reliable SOI process was developed to batch fabricate the WEM resonators, as shown in Figure 7. A (100)-oriented SOI wafer with a 3 μm-thick low resistivity of 0.001~0.005 Ω·cm device layer and 1 μm-thick buried oxide was employed. A 1.3 μm-thick PECVD SiO_2_ was deposited as a mask for silicon etching. Then, the resonator and electrode leads were patterned by inductively coupled plasma (ICP) dry etch. A 70 nm thermal oxide layer was grown to define the transduction gap. A 2 μm highly doped and low stress LPCVD polysilicon was deposited and was patterned to form AC signal electrodes. To reduce the contact resistance, the 400 nm-thick Au/Cr electrode pads were fabricated by an e-beam evaporation and lift-off process. Finally, the resonators were released in a 49% concentrated HF solution. Figure 8 shows the SEM photographs of fabricated WEM resonators.

## 4. Results and Discussion

To comprehensively investigate the performance of resonators, the WEM resonators with different tethers were measured using the Lakeshore CRX-4K vacuum probe platform at a vacuum of 8 × 10^−5^ Torr. A U8032A DC voltage source is used to provide DC bias voltage. An Agilent E5071C network analyzer produces an AC excitation signal. Table 3 shows the *f* and *Q* of the WEM resonators with the straight and combined tethers. The *Q* values of resonators with the combined tethers are superior to those with the straight tethers. As shown in Figure 1, the resonator vibrating in the 1st WEM suffers from large anchor loss due to the large displacement of the tether attachment along the *x*-axis; thus, the *Q* value is only a few thousands. And the output signal of the resonator in the 2nd WEM cannot be detected due to the serious anchor loss caused by the large displacement in the tether ends.

**Figure 9:**
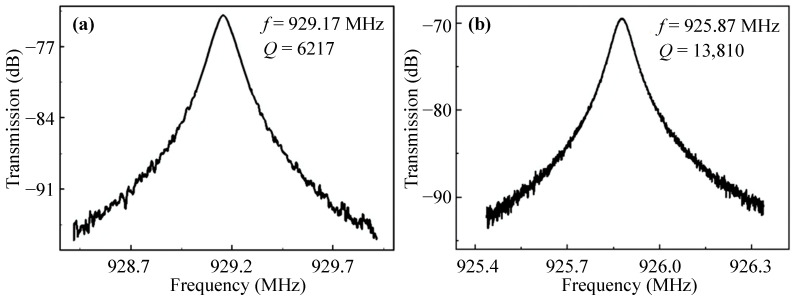
The spectra of the 3rd WEM resonators supported by (**a**) the straight tether and (**b**) the combined tether in vacuum.

**Figure 10:**
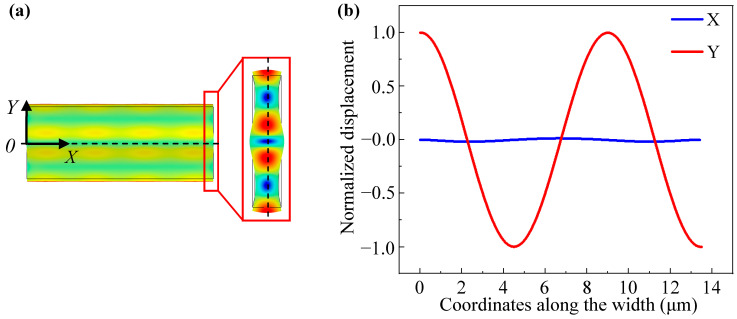
The top and side views of the 3rd WEM mode shape (**a**) and the normalized displacement of the 3rd WEM vs. the coordinates along the width (**b**).

The spectra of the 3rd WEM resonators supported by different tethers are shown in Figure 9, the *Q* value rises from 6217 for the straight tether to 13,810 for the combined one owing to the reduced anchor loss and the *f × Q* product is up to 1.2 × 10^13^. As shown in Figure 10a, the displacement along the width is divided into *X* and *Y* components. To qualitatively describe the displacement, only the middle of the thickness is extracted. As displayed in Figure 10b, the *Y*-component displacement is far less than the *X*-component. As for the *Y*-component, the two sides of the width midpoint vibrate in anti-phase. The vibration at the tether-resonator connection point is weakened for destructive interference. Therefore, the 3rd WEM has relatively less anchor loss than the 1st WEM. Furthermore, as depicted in Figure 6, the AKE damping becomes prominent at around 900 MHz, leading to the measured *Q* values of less than 20,000. Table 4 compares the reported UHF silicon-based resonators. The UHF resonator developed in this work has superior performance.

**Figure 11:**
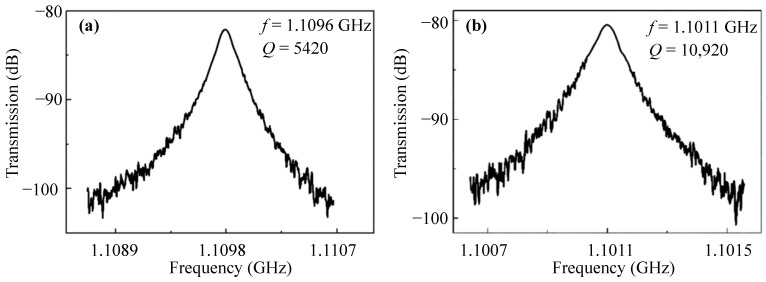
The spectra of the 4th WEM resonators supported by the straight tethers (**a**) and the combined tethers (**b**).

The measured spectra of the 4th WEM resonators with different tethers are displayed in Figure 11. The frequency of the 4th WEM resonator supported by the combined tethers is 1.1 GHz, and the *Q* value is 10,920, which is almost twice as much as those of the conventional WEM resonators. This results from the low amplitude in the tether-plate attachment regions (Figure 1). The AKE damping is more severe than that of the 3rd WEM, and the upper limit of the *Q* value is further reduced to below 20,000. The comparison between the reported GHz resonators and this work is presented in Table 5. The *f × Q* product of the resonators in this work is the highest among the reported bulk acoustic wave (BAW) mode GHz resonators.

The motional impedance can be derived from measured spectra by the following formula:

(14) $$R_{x}=2Z_{0}(10^{-\frac{G_{peak}}{20}}-1),$$

where *Z*_0_ is the characteristic impedance of the network analyzer (50 Ω). *G_peak_* denotes the peak transmission gain of the frequency response spectrum. The measured motional impedance and calculated effective stiffness of the 3rd and 4th WEMs are listed in Table 6. According to (4), for the combined tether, *Q* enhancement and stiffness reduction are effective ways to reduce motional impedance. Compared with the conventional resonators, the motional impedance of the optimized resonator decreases by 33% and 20% in the 3rd and 4th modes, respectively.

The resonator in this work can be easily excited with a low bias voltage of 8 V but has a high impedance of several hundred kΩ, which could be reduced to <100 kΩ with *V_P_* doubled according to Equation (4), as shown in Table 4. The GHz resonators as given in Table 5 can also be driven into vibration with a low bias voltage of 8 V and have an even higher impedance of MΩ; however, it could be reduced to about 250 kΩ with *V_P_* doubled according to Equation (4), comparable to the reported value in Table 5 [14].

The motional impedance of the WEM resonators could be further reduced via enlarging the capacitive area and adopting the resonator array, which has a potential application for wireless communication systems.

## 5. Conclusions

In this work, a GHz capacitively transduced silicon-based WEM resonator with *Q* over 10,000 was demonstrated. A novel combined tether was designed to optimize the *Q* and the motional impedance. The frequency of the 4th WEM is 1.1 GHz, and *Q* is 10,920, corresponding to the promising *f × Q* product of 1.20 × 10^13^, which outperforms the reported GHz resonators. The 3rd WEM resonator of 925.8 MHz was demonstrated with a *Q* of 13,810 and an excellent *f × Q* product of 1.27 × 10^13^. In future work, the plate width can be further shrunk to realize GHz utilizing the 3rd WEM. The performance of the 1st WEM needs to be further optimized to maintain a high *Q* value in all the modes. The combined tether exhibits a blueprint for the resonators with the enhanced *Q*, and reduced motional impedance, which makes the WEM resonators a potential device for the application of wireless communication systems.

## Figures and Tables

**Table 1:** The optimized size of the WEM resonator with the combined tether.

Structure	Parameter	Size (μm)
Plate	Length *L*	38
	Width *W*	13
	Thickness *h*	3
Straight tether	Width *W_s_*_1_	3
	Length *L_s_*_1_	4
Combined tether	Straight beam width *W_s_*_2_	3
	Straight beam length *L_s_*_2_	3
	Trapezoidal frame width *W_x_*	3
	Bottom edge length of trapezoidal frame *W_t_*	15
	Through hole width *W_h_*	2

**Table 2:** Comparison between the simulated frequency and *Q_anchor_* of the resonators with the straight tethers and with the combined tethers.

Type of Tether	Straight Tether		Combined Tether	
Mode order	*f* (MHz)	*Q_anchor_* (×10^4^)	*f* (MHz)	*Q_anchor_* (×10^4^)
1st	316.42	0.52	316.71	0.73
3rd	907.46	7.65	906.01	12.96
4th	1092.94	1.51	1089.59	3.65

**Table 3:** Performance of the WEM resonators of different orders.

Type of Tether	Order	*f* (MHz)	*Q* (×10^4^)	*f × Q*
Straight tether	1	330.44	0.23	7.6 × 10^11^
	3	929.17	0.62	5.7 × 10^12^
	4	1109.65	0.54	5.9 × 10^12^
Combined tether	1	324.33	0.42	1.3 × 10^12^
	3	925.87	1.38	1.2 × 10^13^
	4	1101.10	1.09	1.2 × 10^13^

**Table 4:** The *f × Q* product comparison between the reported UHF silicon-based resonators and this work.

References	[17]	[28]	[29]	[14]	This Work (3rd)
Frequency (MHz)	734.6	651.1	422	764.5	925.87
*Q* (in vacuum)	7890	4700	10,000	17,300	13,810
*f × Q* (×10^12^)	5.79	3.06	4.22	13.23	12.78
*R_x_* (kΩ)	521.4	559.7	297.8	23.7	316.12
Measurement method	Mixing: *V_P_* = 10.5 V *V_LO_* = 7.9 V	Mixing: *V_P_* = 10 V *V_LO_* = 10 V	Mixing: *V_P_* = 7 V *V_LO_* = 6 V	Two-port: *V_P_* = 60 V	Two-port: *V_P_* = 8 V

**Table 5:** The comparison between the reported GHz resonators and this work.

References	[17]	[28]	[14]	This Work (4th)
Frequency (GHz)	1.156	1.523	1.548	1.1011
*Q* (in vacuum)	2683	2800	2900	10920
*f × Q* (×10^12^)	3.10	4.26	4.49	12.02
*R_x_* (kΩ)	2441.9	791.6	300	1059.15
Measurement method	Mixing: *V_P_* = 10.5 V *V_LO_* = 7.9 V	Mixing: *V_P_* = 5 V *V_LO_* = 5 V	Two-port: *V_P_* = 15 V	Two-port: *V_P_* = 8 V

**Table 6:** The comparison of total stiffness of the resonator with different tethers in the 3rd and 4th WEMs.

Type of Tether	Straight Tether			Combined Tether		
Mode order	*R_x_* (kΩ)	*k_eff_* (×10^6^ N/m)	*V_P_* (V)	*R_x_* (kΩ)	*k_eff_* (×10^6^ N/m)	*V_P_* (V)
3rd	473.05	36.19	10	316.12	28.15	8
4th	1333.42	53.01	10	1059.15	44.81	8

## Data Availability

Data is unavailable due to privacy or ethical restrictions.
